# GSA JOURNAL EDITORS PANEL: WHAT HAPPENS AFTER I PRESS SUBMIT? UNDERSTANDING THE JOURNEY OF A MANUSCRIPT

**DOI:** 10.1093/geroni/igad104.0578

**Published:** 2023-12-21

**Authors:** Jessica Kelley

## Abstract

You have finished your great manuscript. Formatted it. Wrote the cover letter. But what happens after you press the “Submit” button? This moderated panel of Editors-in-Chief of five of the GSA journals will talk about all aspects of the review process that lead to a final decision. We will cover topics such as how reviewers are selected, top reasons a manuscript may be rejected without review, and review timetables. We will discuss how Editors use the reviews to make a decision, including how they deal with reviewers who may disagree with each other, or a case where an Editor may disagree with a reviewer. Following the moderated discussion, we will have plenty of time for audience questions.

